# Changing trends in colorectal cancer in the Republic of Korea: contrast with Japan

**DOI:** 10.4178/epih/e2015038

**Published:** 2015-08-17

**Authors:** Minjoo Yoon, Nicholas Kim, Byungho Nam, Jungnam Joo, Moran Ki

**Affiliations:** Department of Cancer Control and Policy, Graduate School of Cancer Science and Policy, National Cancer Center, Goyang, Korea

**Keywords:** Colorectal neoplasms, Incidence, Mortality, Screening, Korea, Japan

## Abstract

Colorectal cancer has a high worldwide incidence. Japan, a country that is geographically and culturally similar to the Republic of Korea (here after Korea), has recently reported a decreasing trend in the incidence of colorectal cancer. However, Korea had the highest incidence of colorectal cancer among Asian countries in 2012. Our aim was to observe the changing trends in incidence and mortality of colorectal cancer in Korea and to compare them to those in Japan. Incidence data were collected from the Korean Central Cancer Registry and mortality data were collected from Korean Statistical Information Service. Incidence and mortality data on colorectal cancer in Japan were acquired from the National Cancer Center in Japan. Age-standardized incidence and mortality rates were determined based on Segi’s world population. Screening data from both countries were collected from the national cancer center in each country. In Korea, the age-standardized incidence rate of colorectal cancer in both sexes was 20.9 to 38.0 per 100,000 from 1999 to 2012 and the rate in males increased more dramatically than in females. In addition, the increase between 2002 and 2012 was first observed in the age group over 40. In Japan, the incidence of colorectal cancer has been more constant over recent years than in Korea. The age-standardized mortality rate of colorectal cancer in both sexes in Korea was 8.5 to 9.3 per 100,000 from 2000 to 2013, and the trend in mortality was constant during this period. In Japan, the mortality rate decreased slightly during the same period. Crude screening rates were increased overall in both Korea and Japan during the period studied. Since the incidence of colorectal cancer has increased in Korea, the control of this cancer is an important public health issue. As Japan has achieved a reduction in colorectal cancer, adjustment of Korea’s current systems for screening and treatment of colorectal cancer according to those of Japan may contribute to improved colorectal cancer control in Korea.

## INTRODUCTION

Globally, colorectal cancer has a high incidence in both males and females [1]. The risk of colorectal cancer varies worldwide due to factors related to Westernized lifestyle [2]. Several developed countries that experienced early Westernization such as the US and Japan show reduced incidence and mortality of colorectal cancer [3-6]. These decreases might be related to screening, during which colorectal status is determined and polyps are eliminated [3]. However, Asian countries that are currently adopting Westernization have experienced an increasing risk of colorectal cancer in the past few decades [7,8].

According to GLOBOCAN 2012, the highest age-standardized incidence rate of colorectal cancer in both sexes was observed in the Republic of Korea (hereafter Korea), compared to other countries in Asia in 2012 (males, 58.7 per 100,000; females, 33.3 per 100,000) [9]. The rates in both sexes in Korea were much higher than that of Japan, in which the largest growth in colorectal cancer incidence was observed from 1998 to 2002 [10]. In addition, the incidence of colorectal cancer in Korea has continued to increase in both sexes, while the mortality rate of colorectal cancer remained constant during the most recent decade [6]. The mortality of colorectal cancer was decreased immediately in Japan after implementing screening in 1992, and the incidence might be expected to continue to steadily decrease [4,6]. Several studies suggest that Japan is the only country in Asia to have achieved a decreased incidence in colorectal cancer in both sexes [6]. Since Japan and Korea are geographically and culturally similar, investigating the colorectal cancer trends in these two countries may be helpful to improve colorectal cancer control in Korea and identify useful lessons from the efforts in Japan.

The aim of the study was to observe the trends in incidence and mortality of colorectal cancer in Korea by sex and age and to compare them to those in Japan, with a view to reducing colorectal cancer risk in Korea.

## MATERIALS AND METHODS

### Incidence, mortality and fatality

Colorectal cancer was classified according to the International Classification of Disease (ICD, 10th revision). The codes for colorectal cancer (C18-C21) refer to the anatomic sites of the colon, rectum, and anus. Incidence data covered the colon to the rectum (C18-C20) and mortality data covered all anatomic sites (C18-C21) studied. Cancer incidence and mortality data were obtained from the Korea Central Cancer Registry and Korean Statistical Information Service, respectively. Incidence data from 1999 to 2012 and mortality data from 2000 to 2013 were collected [11-14]. To compare between Korea and Japan, the age-standardized incidence and mortality rates in both countries were converted based on the world standard population proposed by Segi in 1960 [15]. The Japanese incidence and mortality data were collected from the official website of the National Cancer Center in Japan [16-18].

The age-specific incidence and mortality rates in males and females in Korea were sorted into age distribution groups of 5 years to observe the trends in incidence and mortality rates over a 10 year duration; specifically, from 2002 to 2012 for incidence and from 2003 to 2013 for mortality. The fatality ratio of colorectal cancer in Korea from 2000 to 2012 was determined based on the age-standardized incidence and mortality rates [19].

### Screening

In Korea, the national colorectal cancer screening program began in 2004 [20]. People who receive Medicaid and fall within the lower 50% of the National Health Insurance Corporation (NHIC) premium are the targeted population for the National Cancer Screening Program (NCSP) [21]. Those who are within the upper 50% of the NHIC premium can receive screening with a 20% out-of-pocket payment [21]. People aged above 50 years are eligible for a fecal occult blood test every year as a first national colorectal cancer screening process, and a colonoscopy or double-contrast barium enema test is optionally performed if abnormal symptoms are detected [20]. Screening rates were defined as the rate of screening performed according to the recommendation of the NCSP. The crude Korean screening rates from 2004 to 2014 were collected from the cancer screening report published by the National Cancer Center in Korea in 2014 [22].

In Japan, the target population for the national colorectal cancer screening program is people aged above 40 years who are insured by National Health Insurance. The national screening program starts with an immunochemical fecal occult blood test [16-18,23]. If positive results are detected by this test, other optional tools such as flexible sigmoidoscopy or colonoscopy are then used [24]. Crude screening rates based on this program were available from the National Cancer Center in Japan for the years 2007, 2010, and 2013 [16-18]. The screening participation number in Japan is measured from participants primarily enrolled in the national cancer screening program handled by 3,200 municipalities in 50 prefectures [4,25]. The program follows the guidelines from the Ministry of Health, Labour, and Welfare for managing participants [4]. The crude screening rates in Korea and Japan were used to compare the levels of screening and to observe the relationship between screening and changes in colorectal cancer incidence and mortality.

## RESULTS

### Incidence

**Figure 1.**
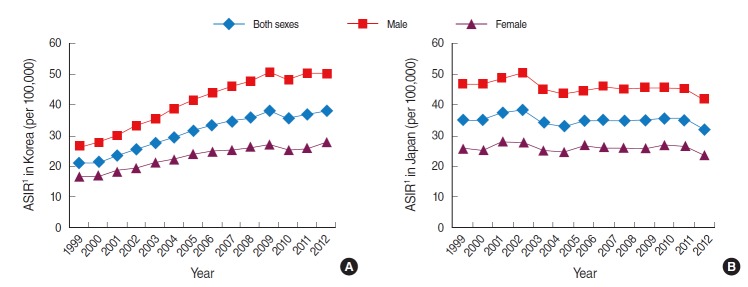
Age-standardized incidence rate (ASIR) of colorectal cancer (ICD-10 codes C18-C20) by sex in Korea (A) and Japan (B), 1999-2012. Data from Korean Statistical Information Service. Number of cancer patients, relative frequency, crude rate, age-adjusted incidence by cancer site and sex from 1999 to 2012 [14]; Cancer Information Service, National Cancer Center, Japan. National estimates of cancer incidence based on cancer registries in Japan (1975-2010) [16]. ICD-10, International Classification of Disease 10th revision. 1Age-standardized to Segi’s 1960 world standard population.

**Figure 2.**
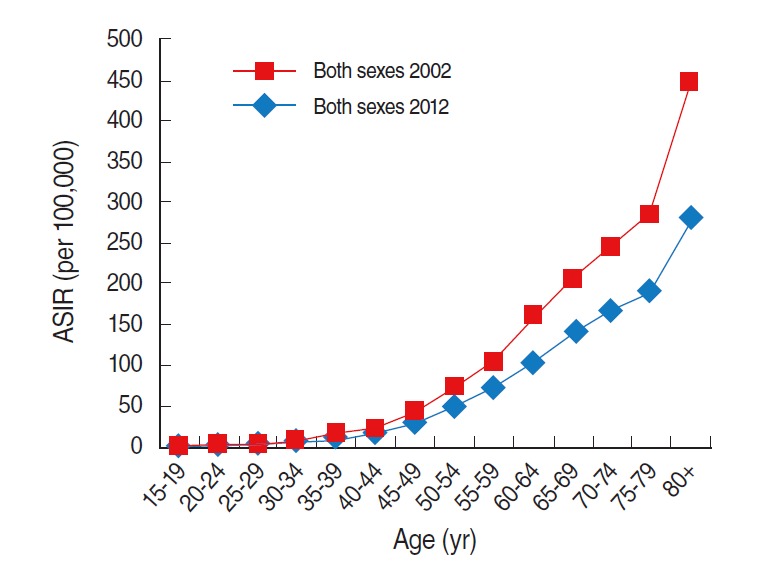
Age-specific incidence rate (ASIR) of colorectal cancer (ICD-10 codes C18-C20) in Korea in 2002 and 2012. Data from Korean Statistical Information Service. Number of cancer patients, relative frequency, crude rate, age-adjusted incidence by cancer site and sex from 1999 to 2012 [14]. ICD-10, International Classification of Disease 10th revision.

In Korea, the age-standardized incidence rate of colorectal cancer in both sexes was 20.9 per 100,000 persons in 1999 and 38.0 in 2012 (Table 1). In males, the age-standardized incidence rate was 26.7 in 1999, and this increased to 50.3 in 2012 (Table 1). The rate in females was 16.9 in 1999, and this increased to 27.7 in 2012 (Table 1). Compared to females, the incidence in males increased dramatically from 1999 to 2012 (Figure 1). The leap in incidence between 2002 and 2012 was observed in individuals aged 40 years or above, and the highest leap in the incidence rate among the age groups was in the age group above 80 years (Table 2, Figure 2).

In Japan, the age-standardized incidence rate of colorectal cancer did not dramatically change from 1999 to 2012 (Figure 1). The age-standardized incidence rate in both sexes changed from 35.6 in 1999 to 32.2 in 2012; in males from 47.1 to 42.1 and in females from 26.1 to 23.5 (Table 1). Although the trend in cancer incidence in Korea was much higher than that in Japan from 1999 to 2012, the age-standardized rates of cancer incidence in Japan were higher than in Korea for both sexes until 2007 (Table 1).

### Mortality

**Figure 3.**
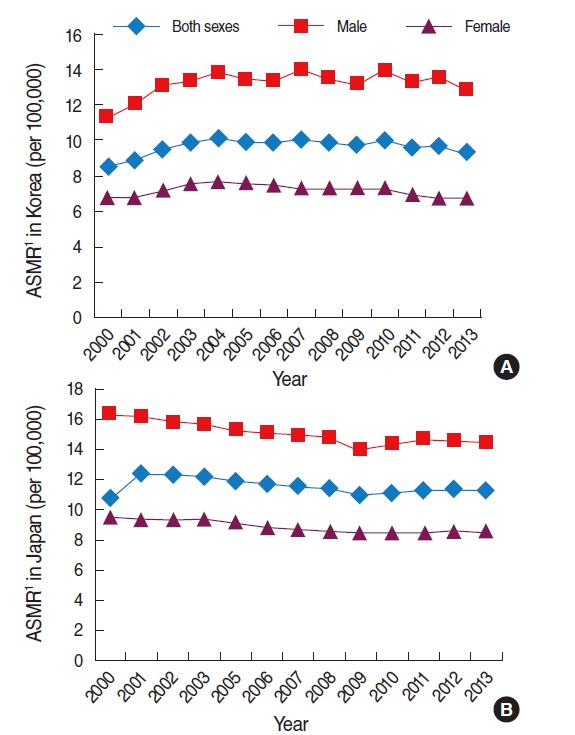
Age-standardized mortality rate (ASMR) of colorectal cancer (ICD-10 codes C18-C21) in Korea (A) and Japan (B), 2000 to 2013. Data from Korean Statistical Information Service Office. Death causes by 5-year age group and gender, mortality rates (1983-2013) [13]; Cancer Information Service, National Cancer Center, Japan. Cancer mortality from vital statistics in Japan (1958-2013) [17]. ICD-10, International Classification of Disease 10th revision. 1Age-standardized to Segi’s 1960 world standard population.

**Figure 4.**
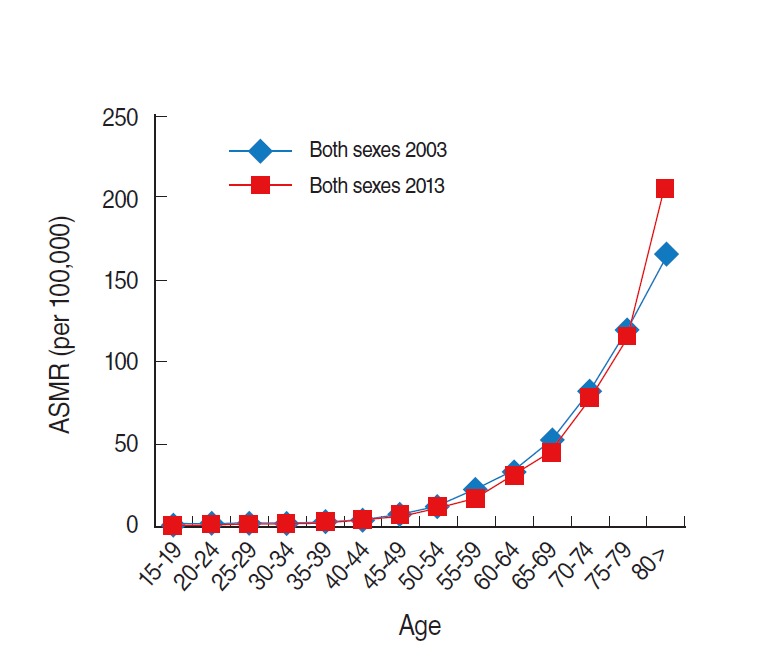
Age-specific mortality rate (ASMR) of colorectal cancer (ICD-10 codes C18-C21) in Korea in 2003 and 2013. Data from Korean Statistical Information Service Office. Death causes by 5-year age group and gender, mortality rates (1983-2013) [13]. ICD-10, International Classification of Disease 10th revision.

In Korea, the age-standardized mortality rate in males was higher than that in females, and there was no significant increase in these rates from 2000 to 2013 (Figure 3). The age-standardized mortality rate in both sexes changed from 8.5 to 9.3; 11.3 to 12.9 in males and 6.8 to 6.7 in females (Table 1). In both sexes, the age group above 80 years had the highest increase among all age groups from 2003 to 2013, whereas slight decreases in mortality rates were observed in the age groups between 50 and 79 years (Table 2, Figure 4).

In Japan, the age-standardized mortality rate of colorectal cancer decreased slightly and remained constant in all groups from 2000 to 2013 (Figure 3). The age-standardized mortality rate in both sexes changed from 10.7 to 11.2 between these years; from 16.4 to 14.4 in males and from 9.4 to 8.5 in females (Table 1). However, the age-standardized mortality rate in Japan had been higher than Korea in both sexes during the periods examined except for in 2004 (Table 1). The common factor in the mortality trends in both countries was that the trend was not increasing.

### Fatality

**Figure 5.**
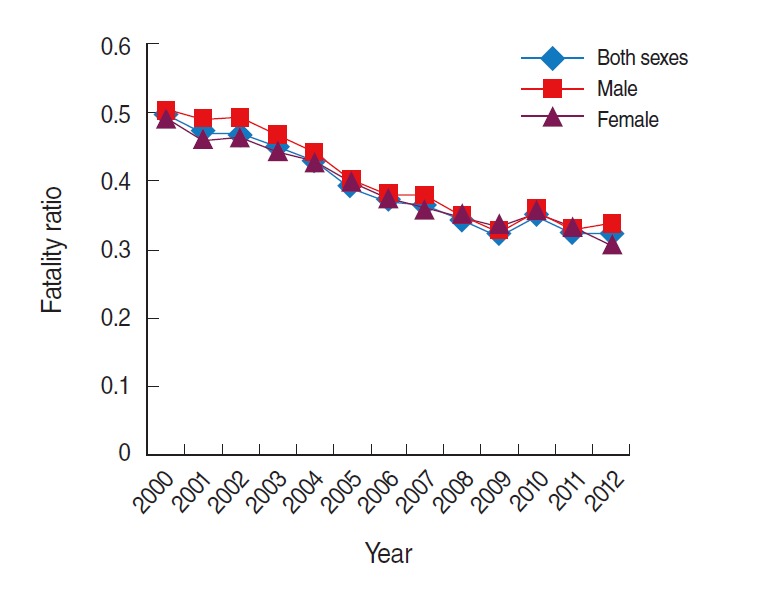
Fatality ratio of colorectal cancer (ICD-10 codes C18-C20) in Korea, 2000 to 2012. Data from Korean Statistical Information Service Office. Death causes by 5-year age group and gender, mortality rates (1983-2013) [13]; Korean Statistical Information Service. Number of cancer patients, relative frequency, crude rate, age-adjusted incidence by cancer site and sex from 1999 to 2012 [14]. ICD-10, International Classification of Disease 10th revision.

The fatality ratio was obtained from the data on age-standardized incidence and mortality rates in Korea from 2000 to 2012, and a decreasing trend in the fatality ratio was observed during the study period (Table 3, Figure 5).

### Screening

**Figure 6.**
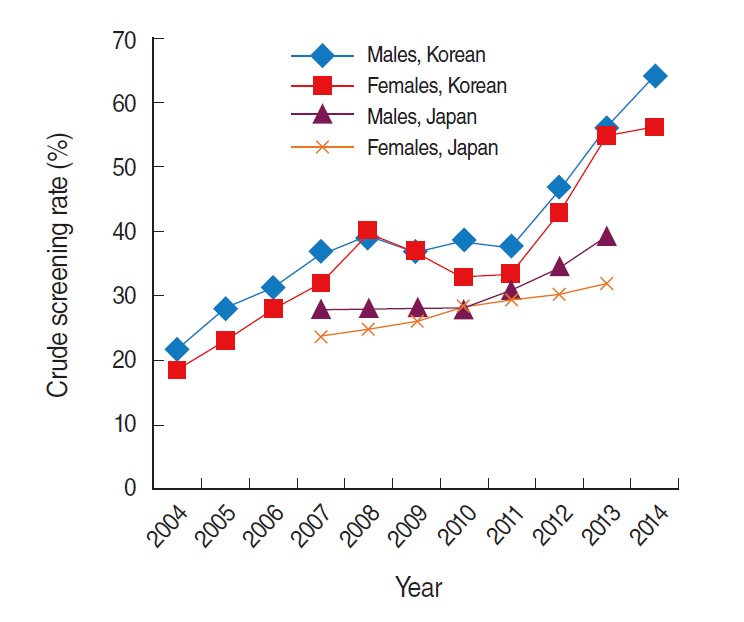
Crude screening rate for colorectal cancer (ICD-10 codes C18-C20) in Korea (2004 to 2014) and Japan (2007, 2010, and 2013). Data from Cancer Information Service, National Cancer Center, Japan. Cancer statistics in Japan ‘14: cancer screening rate (2007, 2010, 2013) [18]; National Cancer Center. 2014 Annual survey report of cancer screening performance in Korea. Goyang: National Cancer Center; 2014 [22]. ICD-10, International Classification of Disease 10th revision.

The crude screening rates in Korean males and females were 21.5% and 18.5%, respectively, in 2004, and both rates fluctuated until 2011 (Table 4, Figure 6). The rates in males and females increased to 64.1% and 56.2% in 2014 (Table 4). The crude screening rates in Japanese males and females were 27.9% and 23.7%, respectively, in 2007, 28.1% and 28.3% in 2010, and 39.3% and 32.1% in 2013 (Table 4).

## DISCUSSION

Based on this study, the age-standardized incidence rates of colorectal cancer are increasing and the mortality rates are constant in Korea. The fatality ratio in Korea appears to have decreased during the period studied. This may indicate that precursor cancers or polyps occurred and were detected while the mortality rates remained constant. In contrast, incidence and mortality rates in Japan showed decreasing trends. Previous studies have suggested that the rapid increase in colorectal cancer incidence and mortality in both sexes in Japan has started to slow down and to stabilize [6,26]. The changes in the incidence, mortality and fatality of colorectal cancer could be affected by screening systems, which are used for the early detection of colorectal cancer [6]. Implementing screening could lead to a constant mortality rate and a decreasing fatality ratio [27]. Although screening may lead to increasing incidence rates by detecting precursor lesions related to colorectal cancer, a reduction in incidence could ultimately be observed [3,27]. A national colorectal cancer screening program has been implemented since 1992 in Japan, and this may play a crucial role in colorectal cancer control in that country [4,6].

Notably, the recommended screening ages are above 50 years in Korea and above 40 years in Japan [28,29]. Korea, like Japan, has a high proportion of elderly people in its society; however, the recommended age of colorectal cancer screening in Korea is higher compared to that in Japan. We noted that the leap in colorectal cancer incidence between 2002 and 2012 was first observed in individuals aged above 40 years. In addition, colorectal cancer incidence in younger age groups aged lower than 50 years is increasing in Korea [30]. Therefore, colorectal cancer incidence could be reduced if the adjustment of recommended screening ages were considered and further studies were performed.

The main limitation of this study was the lack of screening data from Japan; the available data covered only three years: 2007, 2010, and 2013. In addition, screening rates in both countries were crude rates because of the differences in population size between the two countries. Therefore, an exact comparison of screening rates between Korea and Japan was difficult to perform.

In conclusion, the trends in colorectal cancer incidence and mortality in Korea that we have measured indicate that controlling the increasing incidence of cancer in Korea is important. Improvement in the current colorectal cancer detection and treatment systems in Korea, taking into consideration those already in place in Japan, a country which has achieved a decrease in the incidence and mortality of colorectal cancer ahead of Korea, may contribute to cancer control in Korea.

## Figures and Tables

**Table 1.** Age-standardized incidence (1999-2012) and mortality (2000-2013) rates of colorectal cancer^1^ by sex in Korea and Japan

	Korea						Japan					
	C18-C20			C18-C21			C18-C20			C18-C21		
	Incidence rate (per 100,000)^2^			Mortality rate (per 100,000)^2^			Incidence rate (per 100,000)^2^			Mortality rate (per 100,000)^2^		
Year	Both sexes	Male	Female	Both sexes	Male	Female	Both sexes	Male	Female	Both sexes	Male	Female
1999	20.9	26.7	16.9	-	-	-	35.6	47.1	26.1	-	-	-
2000	21.6	27.8	17.0	8.5	11.3	6.8	35.3	47.0	25.4	10.7	16.4	9.4
2001	23.5	30.2	18.5	8.9	12.0	6.8	37.6	49.0	28.0	12.4	16.2	9.4
2002	25.3	33.3	19.4	9.5	13.1	7.2	38.3	50.7	27.8	12.2	15.8	9.3
2003	27.5	35.8	21.2	9.9	13.4	7.6	34.5	45.1	25.5	12.2	15.7	9.4
2004	29.3	38.7	22.2	10.1	13.8	7.7	33.4	43.6	24.7	8.7	15.8	9.3
2005	31.7	41.7	23.8	10.0	13.4	7.6	35.1	44.7	26.9	11.9	15.3	9.1
2006	33.3	44.0	24.9	9.9	13.4	7.5	35.3	45.9	26.0	11.6	15.1	8.8
2007	34.6	46.1	25.5	10.1	14.0	7.3	34.9	45.3	25.8	11.6	15.0	8.7
2008	35.9	48.0	26.2	9.9	13.5	7.4	35.1	45.8	25.9	11.4	14.8	8.5
2009	37.9	50.9	27.4	9.7	13.2	7.3	35.6	45.8	26.9	10.9	14.0	8.4
2010	35.7	48.2	25.3	10.0	13.9	7.3	35.3	45.4	26.7	11.1	14.3	8.4
2011	37.1	50.2	26.0	9.6	13.3	7.0	-	-	-	11.3	14.7	8.4
2012	38.0	50.3	27.7	9.7	13.6	6.8	32.2	42.1	23.5	11.3	14.6	8.6
2013	-	-	-	9.3	12.9	6.7	-	-	-	11.2	14.4	8.5

Source from Korean Statistical Information Service Office. Death causes by 5-year age group and gender, mortality rates (1983-2013) [13]; Korean Statistical Information Service. Number of cancer patients, relative frequency, crude rate, age-adjusted incidence by cancer site and sex from 1999 to 2012 [14]; Cancer Information Service, National Cancer Center, Japan. National estimates of cancer incidence based on cancer registries in Japan (1975-2010) [16]; Cancer Information Service, National Cancer Center, Japan. Cancer mortality from Vital Statistics in Japan (1958-2013) [17]. ICD-10, International Classification of Disease 10th revision.

1 ICD-10 codes of C18-C20 and C18-C21.

2 Age-standardized rate per 100,000 using Segi’s world standard population of 1960. Age-standardization of incidence and mortality adapted from Bray [4].

**Table 2.** Age-specific incidence (2002 and 2012) and mortality (2003 and 2013) rates of colorectal cancer^1^ in Korea

Age (yr)	C18-C20		C18-C21	
	Incidence rate (per 100,000)		Mortality rate (per 100,000)	
	2002	2012	2003	2013
15-19	0.2	0.3	-	-
20-24	0.6	0.9	16.4	9.4
25-29	1.8	2.6	16.2	9.4
30-34	3.9	7.0	15.8	9.3
35-39	8.4	14.0	15.7	9.4
40-44	15.8	22.4	15.8	9.3
45-49	27.0	40.6	15.3	9.1
50-54	47.5	72.0	15.1	8.8
55-59	73.2	104.5	15.0	8.7
60-64	103.7	160.8	14.8	8.5
65-69	142.8	206.4	14.0	8.4
70-74	166.8	244.5	14.3	8.4
75-79	190.7	284.9	14.7	8.4
80+	280.6	447.8	14.6	8.6

Source from Korean Statistical Information Service Office. Death causes by 5-year age group and gender, mortality rates (1983-2013) [13]; Korean Statistical Information Service. Number of cancer patients, relative frequency, crude rate, age-adjusted incidence by cancer site and sex from 1999 to 2012 [14].

ICD-10, International Classification of Disease 10th revision.

1 ICD-10 codes of C18-C20 and C18-C21.

**Table 3.** Fatality ratios of colorectal cancer^1^ in Korea (2000-2012)

Year	Both sexes	Male	Female
2000	0.5	0.5	0.5
2001	0.5	0.5	0.5
2002	0.5	0.5	0.5
2003	0.4	0.5	0.4
2004	0.4	0.4	0.4
2005	0.4	0.4	0.4
2006	0.4	0.4	0.4
2007	0.4	0.4	0.4
2008	0.3	0.4	0.4
2009	0.3	0.3	0.3
2010	0.3	0.4	0.4
2011	0.3	0.3	0.3
2012	0.3	0.3	0.3

Source from Korean Statistical Information Service Office. Death causes by 5-year age group and gender, mortality rates (1983-2013) [13]; Korean Statistical Information Service. Number of cancer patients, relative frequency, crude rate, age-adjusted incidence by cancer site and sex from 1999 to 2012 [14].

ICD-10, International Classification of Disease 10th revision.

1 ICD-10 codes of C18-C20.

**Table 4.** Crude screening rates of colorectal cancer (ICD-10 codes C18-C20) in Korea (2004-2014) and Japan (2007, 2010, and 2013) by sex

Year	Korean (%)		Japanese (%)	
	Male	Female	Male	Female
2004	21.5	18.5	-	-
2005	28	23	-	-
2006	31.2	27.8	-	-
2007	36.9	31.8	27.9	23.7
2008	39.3	40.1	-	-
2009	36.5	36.8	-	-
2010	38.5	32.7	28.1	28.3
2011	37.4	33.3	-	-
2012	46.7	42.8	-	-
2013	56.3	54.9	39.3	32.1
2014	64.1	56.2	-	-

Source from Cancer Information Service, National Cancer Center, Japan. Cancer statistics in Japan ‘14: cancer screening rate (2007, 2010, 2013) [18]; National Cancer Center. 2014 Annual survey report of cancer screening performance in Korea. Goyang: National Cancer Center; 2014 [22].

ICD-10, International Classification of Disease 10th revision.
